# Significant oligodendrocyte progenitor and microglial cell death is a feature of remyelination following toxin-induced experimental demyelination

**DOI:** 10.1093/braincomms/fcae386

**Published:** 2025-01-17

**Authors:** Hallie Gaitsch, Peggy Assinck, Penelope Dimas, Chao Zhao, Laura Morcom, David H Rowitch, Daniel S Reich, Robin J M Franklin

**Affiliations:** Wellcome-MRC Cambridge Stem Cell Institute, University of Cambridge, Cambridge CB2 0AW, UK; Translational Neuroradiology Section, National Institute of Neurological Disorders and Stroke, National Institutes of Health, Bethesda, MD 20892, USA; Wellcome-MRC Cambridge Stem Cell Institute, University of Cambridge, Cambridge CB2 0AW, UK; Altos Labs, Cambridge Institute of Science, Cambridge CB21 6GQ, UK; Wellcome-MRC Cambridge Stem Cell Institute, University of Cambridge, Cambridge CB2 0AW, UK; Altos Labs, Cambridge Institute of Science, Cambridge CB21 6GQ, UK; Wellcome-MRC Cambridge Stem Cell Institute, University of Cambridge, Cambridge CB2 0AW, UK; Altos Labs, Cambridge Institute of Science, Cambridge CB21 6GQ, UK; Wellcome-MRC Cambridge Stem Cell Institute, University of Cambridge, Cambridge CB2 0AW, UK; Department of Paediatrics, University of Cambridge, Cambridge CB2 0QQ, UK; Wellcome-MRC Cambridge Stem Cell Institute, University of Cambridge, Cambridge CB2 0AW, UK; Department of Paediatrics, University of Cambridge, Cambridge CB2 0QQ, UK; Translational Neuroradiology Section, National Institute of Neurological Disorders and Stroke, National Institutes of Health, Bethesda, MD 20892, USA; Wellcome-MRC Cambridge Stem Cell Institute, University of Cambridge, Cambridge CB2 0AW, UK; Altos Labs, Cambridge Institute of Science, Cambridge CB21 6GQ, UK

**Keywords:** cell death, microglia, oligodendrocyte, oligodendrocyte progenitor cell, remyelination

## Abstract

The extent to which glial cell turnover features in successful remyelination is unclear. In this study, the rat caudal cerebellar peduncle-ethidium bromide lesion model was used to profile oligodendroglial and microglial/macrophage cell death and proliferation dynamics over the course of repair. Lesioned and control tissue was co-labelled with antibody markers for cell identity, proliferation, and apoptosis (TUNEL assay), then imaged at full thickness using confocal microscopy and quantified using custom CellProfiler pipelines. Early remyelination time points were marked by an increased density of total proliferating cells, including oligodendrocyte progenitor cells. Late remyelination time points featured increased TUNEL+ oligodendrocyte progenitor cells: however, most TUNEL+ cells within remyelinating lesions were Iba1+ microglia/macrophages. These results indicate that repairing lesions are characterized by a high degree of glial cell death and suggest that monitoring cell death-related by-products might have clinical value in the setting of remyelination.

## Teaser

Gaitsch *et al*. used an experimental model of toxin-induced demyelination to profile glial cell death/proliferation dynamics over the course of remyelination. They report that significant oligodendrocyte progenitor and microglial cell death are features of remyelination. These results may guide future investigation of glial cell death by-products as remyelination biomarkers.

## Graphical Abstract

**Graphical Abstract:**
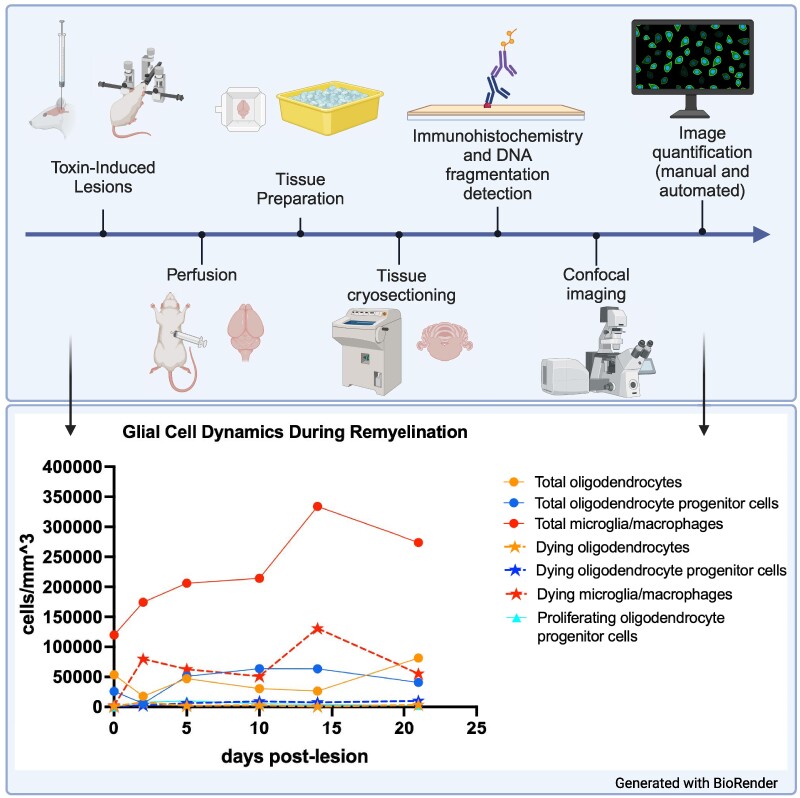

## Introduction

When primary demyelination occurs in the CNS, axonal functional impairment and damage can result, likely driving the development of neurological deficits corresponding to the function of neurons in the affected location.^1-3^ Remyelination is a regenerative process resulting from the successful migration, proliferation, and differentiation of oligodendrocyte progenitor cells (OPC) into myelinating oligodendrocytes (OL) that can repair demyelinated lesions.^4^ The fate of a neuron following demyelinating injury is heavily influenced by the success or failure of remyelination.^5,6^ Remyelination failure leaves denuded axons vulnerable to damage and contributes to the clinical progression of demyelinating diseases of the CNS such as multiple sclerosis.^7^

Recently, several potential remyelinating agents have proceeded to clinical trials.^8-10^ However, methods for accurately and non-invasively assessing changes in remyelination status remain limited.^9,11^ Functional measures (e.g. visual evoked potentials) only measure the effects of demyelination/remyelination along specific neural pathways, MRI techniques (e.g. magnetisation transfer ratio) still face obstacles with respect to remyelination sensitivity and specificity, and clinical disability scales (e.g. expanded disability status scale) are too crude to measure change over the short duration of most clinical trials.^12^ Thus, there would be substantial clinical utility in an alternative or complementary method to detect remyelination, such as a blood-based biomarker to indicate that myelin repair has taken place. In addition to its value as an outcome measure for clinical trials of putative remyelinating therapies, reliable biomarkers of remyelination might be useful to assess baseline remyelination capacity to determine whether to initiate treatment and to measure treatment response in patients already undergoing regenerative therapy.

By-products released by the cells involved in myelin regeneration may be key to future non-invasive detection of this process. For example, cell turnover via apoptosis is the primary mechanism through which cell-free DNA (cfDNA)—short, mononucleosome, or oligonucleosome-length sections of DNA that are increasingly being used as substrates for ‘liquid biopsy’ approaches—is released in both healthy and disease states.^13^ Periods of proliferation have also been associated with the release of nucleic acid by-products into the bloodstream. To this end, potential remyelination-associated biomarker targets (cell-free nucleic acids, secreted proteins, metabolites, and extracellular vesicles) may be elucidated by profiling the cellular dynamics surrounding the remyelination period to identify potential cellular sources.

In this study, oligodendroglial lineage cell death and proliferation dynamics, as well as microglia/macrophage cell death dynamics, were profiled over the course of remyelination. Here, we test the hypothesis that cell death accompanies or follows progenitor cell proliferation events in the context of remyelination.

## Materials and methods

### Animal welfare and husbandry

Animals were housed under standard laboratory conditions with uninterrupted access to food and water in groups of 2–3, randomly allocated to experimental groups based on their sequence of arrival to the facility. A total of 29 rats were used, including five unlesioned controls. Sample size was decided based on prior studies using this model.^14-16^ All rats in this study were female Sprague–Dawley (Charles River), aged 8–11 weeks. All animals underwent a weeklong facility acclimatisation period prior to surgery. Animal pre-operative, intraoperative, and post-operative care included administration of subcutaneous fluids, heat, and analgesics. All animal procedures were performed in compliance with institutional guidelines and United Kingdom Home Office regulations under PPL PC0C0F291.

### Rat caudal cerebellar peduncle-ethidium bromide lesioning

Ethidium bromide (EB) is a DNA alkylating agent that induces astrocyte and oligodendroglial cell loss when introduced into the CNS white matter, leading to blood–brain barrier disruption and demyelination while sparing axons.^16,17^ In this well-established model, healthy, young rats experience complete spontaneous remyelination by 4 weeks post-injury.^14^ A 0.01% EB solution (Thermo Fisher Scientific) was prepared. Bilateral demyelinating lesions were induced by stereotaxic injection of 4 µl 0.01% EB into the cerebellar peduncle (CCP) white matter tracts. Stereotaxic coordinates were selected based on the age of the animals: from bregma, AP −10.4 mm, ML ±2.6 mm, and DV −7.4 mm. EB was injected at 1 µl/min into both CCPs consecutively using a Hamilton syringe, then the needle was kept in place for 4 min to minimize reflux. Post-operative checks were performed twice daily for the first 3 days following surgery. Post-operative checks included assessment of rodent pain behaviours, signs of potential neurological dysfunction (including head tilt and lack of coordination), surgical wound healing, and administration of analgesics. See Supplementary Fig. S1 for experimental outline and model characteristics.

### Tissue preparation

Lesioned rats were split into five groups (4–5 animals/group) based on their perfusion time point (days post-lesion, dpl): Group 1 (2 dpl), Group 2 (5 dpl), Group 3 (10 dpl), Group 4 (14 dpl), and Group 5 (21 dpl). Rats were perfused with 100–200 ml PBS, followed by 500 ml fresh 4% paraformaldehyde (PFA) for tissue fixation. Brains were dissected and immersed in 4% PFA overnight. Each day thereafter, tissue specimens were transferred to sucrose solutions of increasing concentrations (12%, 18%, and 24% sucrose in PBS), then frozen in OCT on dry ice and stored at −80°C. Rat cerebella were cryosectioned (Leica Biosystems) at 12-μm thickness. Lesions were identified by wetting sections with PBS and observing disrupted cellular morphology under a light microscope and/or by staining tissues with Hoechst (nuclei) and localising regions of tissue disruption under a fluorescent microscope. If no clear lesion could be identified at this stage, animals were excluded from further analysis. Slides containing three sequential fixed-frozen sections were prepared and stored at −80°C. Unlesioned CCP tissue from control animals was prepared in the same way. Slides at the core of each lesion were selected for subsequent staining/imaging. The following numbers of animals were analysed for each group: Control (*n* = 5), Group 1 (*n* = 4), Group 2 (*n* = 3); Group 3 (*n* = 5); Group 4 (*n* = 3); Group 5 (*n* = 5).

### Tissue staining and immunofluorescence

#### TUNEL assay and nuclear staining

In situ apoptosis detection was achieved using the TUNEL assay (terminal deoxynucleotidyl transferase (TdT) dUTP nick end labelling; Click-iT Plus TUNEL Assay Kit, Alexa Fluor 488, Thermo Fisher Scientific) to detect fragmented DNA that is a hallmark of the later stages of programmed cell death. The kit protocol was modified to optimize downstream antibody binding for co-immunofluorescence. In brief, heat-induced antigen retrieval was carried out by immersing slides in 80–90°C citrate buffer [pH 6, diluted 1:10 in deionized (DI) water] for 8 min. Tissue fixation was carried out by immersing slides in 4% PFA for 15 min at 37°C, then washing with PBS. Tissue was permeabilized by incubating slides with a proteinase K solution (1:25 in PBS) for 15 min at room temperature. Slides were washed in PBS, then re-fixed by incubating with 4% PFA for 5 min at 37°C, and then washed with PBS and rinsed with DI water. For a positive control, fixed/permeabilized, unlesioned tissue was incubated with DNase I in DNase I reaction buffer (Thermo Fisher Scientific) for 30 min at room temperature to induce DNA strand breaks, then rinsed with DI water. Next, slides were incubated with TdT reaction buffer for 10 min at 37°C. After removing the TdT reaction buffer, slides were incubated with TdT reaction mixture (TdT reaction buffer, EdUTP, and TdT enzyme) for 60 min at 37°C. Following incubation, slides were rinsed in DI water, washed with 10% normal donkey serum (NDS, in 0.1% PBS-Triton), and rinsed with PBS. The reaction to fluorescently detect EdUTP incorporated into fragmented DNA was completed by incubating slides with a TUNEL reaction cocktail (supermix containing Alexa Fluor picolyl azide dye and a copper catalyst) for 30 min at 37°C. Excess reaction cocktail was removed, and slides were washed with 10% NDS (in PBS), then rinsed with PBS. After completion of the TUNEL assay, slides were stained with Hoechst fluorescent stain (1:10 000 in 0.1% PBS-T, Thermo Fisher Scientific) to visualize nuclei, then washed with PBS. See Supplementary Fig. S1 for experimental outline and model characteristics.

#### Immunofluorescence

Following completion of the TUNEL assay and Hoechst staining, tissue was blocked with 10% NDS (in DI water) for 30 min at room temperature. Primary and secondary antibody mixtures were prepared in 0.1% PBS-Triton at the concentrations listed in Supplementary Table S1. Slides were incubated with primary antibody mixtures overnight at 4°C. The next day, slides were assessed for signs of dehydration and washed three times for 10 min each in PBS with gentle agitation to remove excess primary antibody. Slides were incubated with secondary antibody for 2 h at room temperature. Slides were washed three times for 10 min each in PBS with gentle agitation to remove excess secondary antibody, then mounted in Fluoromount-G (Thermo Fisher Scientific) and coverslipped.

### Confocal microscopy

Stained tissue sections were imaged at full thickness using a Leica SP8 laser confocal microscope (Leica Microsystems). The ‘Navigator’ scanning feature was used to capture the entire lesion or control CCP region of interest (ROI). Imaging configuration and acquisition parameters, including gain, offset, and laser percentage, were kept consistent between slides to allow for accurate quantification and comparison. Universal imaging settings were chosen for each stain/antibody panel based on pilot imaging of several slides. The Z-stack range was set to capture the full thickness of each section with a Z-step size of 0.5 µm. For each slide, tissue sections were imaged sequentially from top to bottom and left to right. Images of three regions of interest (control CCP or lesioned CCP) were acquired for each control and experimental animal. Tissue section regions of interest were skipped if the majority of the lesion was covered in a tissue fold or bubble. Following image acquisition, 40× Navigator image tiles were merged and exported as .lif files for image quantification.

### Image quantification

Confocal images were manually checked for imaging and tissue artefacts. Several Z-projections of merged images from each staining panel were manually quantified in Fiji/ImageJ^18^ in a blinded manner (PA and PD blinded image names and group assignments during manual counting by HG) using the ImageJ Cell Counter plugin to check for consistent staining morphology between images/animals and to establish universal brightness threshold values for the entire set of images for each stain/antibody panel. Prior to final quantification, image pre-processing was completed in a semi-automated fashion using ImageJ/Fiji macroscripts for each stain/antibody panel. Universal brightness/contrast settings were applied to each channel and ROI areas were calculated. Pre-processed images were fed into CellProfiler,^19^ a free, open-source software that was used to automatically calculate cell counts. Custom CellProfiler pipelines were created for each stain/antibody panel. Further details about manual versus automatic quantification methods and CellProfiler pipeline modules are presented in Supplementary Figs. S2 and S3.

### Statistical analysis

All statistical analysis was performed using GraphPad Prism 8 software.^20^ Results are presented as mean ± standard deviation. Differences between groups were evaluated for significance by one-way ANOVA with Dunnett’s test for multiple comparisons. Linear regression was used to model the density of TUNEL+ OLs and OPCs over time. Probability (*P*) values were considered statistically significant at ≤0.05.

## Results

The time points chosen for rat perfusion encompassed the time course from peak demyelination (Day 2) to near-complete remyelination (Day 21), as outlined in prior studies describing regeneration kinetics in this model.^21-23^

### Early remyelination time points feature an increased density of total proliferating cells, including OPCs

**Figure 1:**
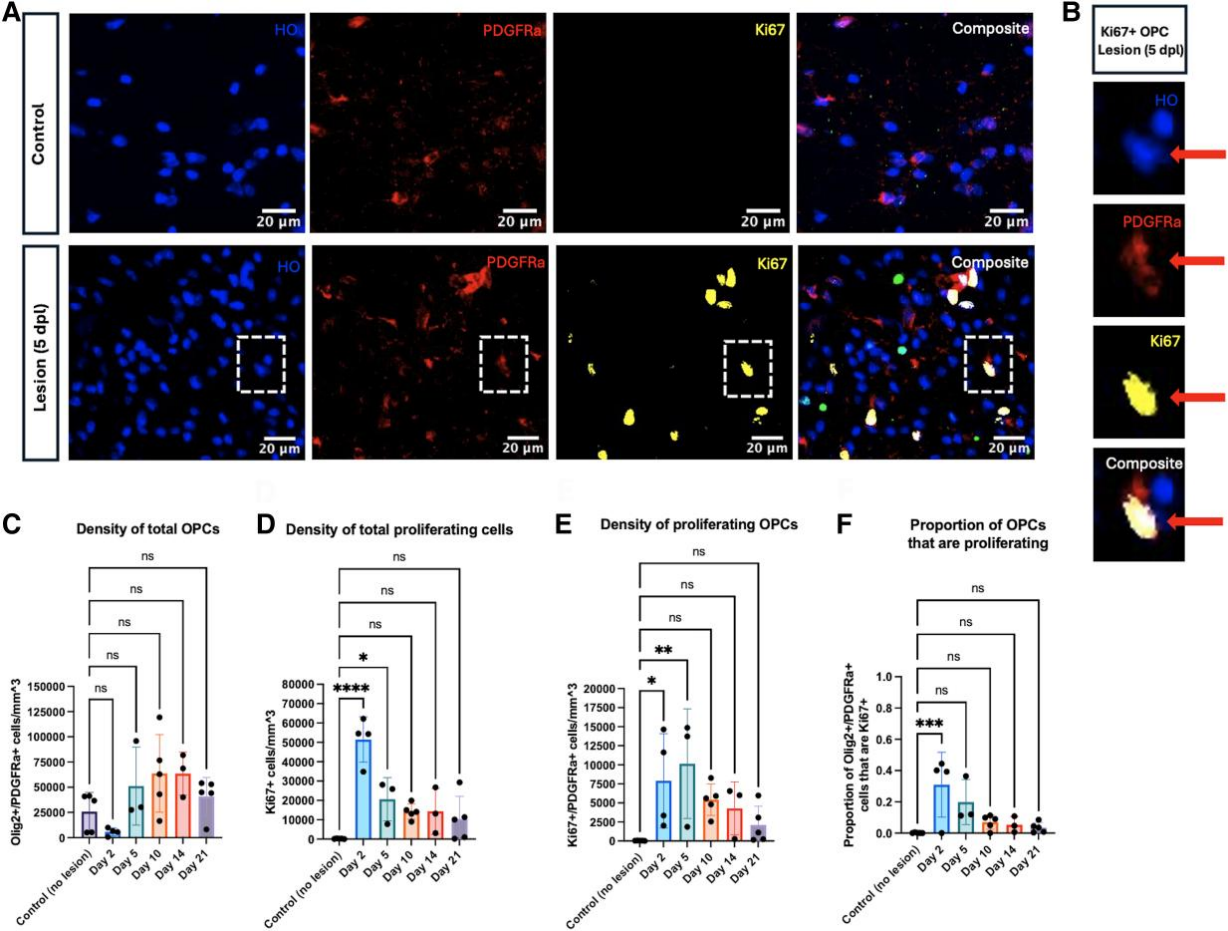
**Early remyelination time points are marked by an increased density of total proliferating cells, including OPCs.** (**A**) Representative immunofluorescence images of control and 5-day-old lesion tissue. Dashed box indicates cell shown in (**B**). Hoechst (HO, blue); PDGFRα (red); Ki67 (yellow); composite (white). (**B**) Representative immunofluorescence images of a single Ki67+ OPC. (**C**) The density of total OPCs was quantified for each repair time point. (**D**) The density of total proliferating cells was quantified for each repair time point. (**E**) The density of proliferating OPCs (Ki67+/PDGFRα+ cells) was quantified for each repair time point. (**F**) The proportion of proliferating OPCs was quantified for each repair time point. Each individual data point represents the average across three regions of interest for a single animal. Bars represent mean ± SD. *P < 0.05, **P < 0.01, ***P < 0.001, and ****P < 0.0001 as determined by one-way ANOVA (**C–F**; control, *n* = 5; Day 2, *n* = 4; Day 5, *n* = 3; Day 10, *n* = 5; Day 14, *n* = 3; Day 21, *n* = 5), comparing each experimental group to the control group and applying Dunnett’s method for multiple comparisons. Abbreviation: dpl, days post-lesion.

Immunostaining for PDGFRα and Ki67 indicated the presence of proliferating OPCs within the lesion boundary (as defined by the region of disrupted tissue morphology observed after Hoechst staining) (Fig. 1A and B). No apparent difference was observed across time points in the mean density of total OPCs (Fig. 1C). There was an increase in the density of total proliferating cells at Day 2 (*P* < 0.0001) and Day 5 (*P* = 0.0251) compared to controls (Fig. 1D). The density of total proliferating cells was not different compared to baseline at the later time points (Days 10, 14, and 21). Within this population of proliferating cells, the density of proliferating OPCs (Ki67+/PDGFRα+) displayed a similar pattern of being increased compared to control at early time points (control versus Day 2: *P* = 0.0287; control versus Day 5: *P* = 0.0087) with no difference compared to control levels by Day 10 (Fig. 1E). At Day 2, approximately one-third of total OPCs remaining at the lesion site were proliferating (mean = 0.3095), an increase compared to baseline OPC proliferation levels (*P* = 0.0009) (Fig. 1F). Beyond the Day 2 time point, no difference in the proportion of proliferating OPC was observed compared to controls.

### A remyelination-associated peak in TUNEL+ cells occurs during late repair

**Figure 2:**
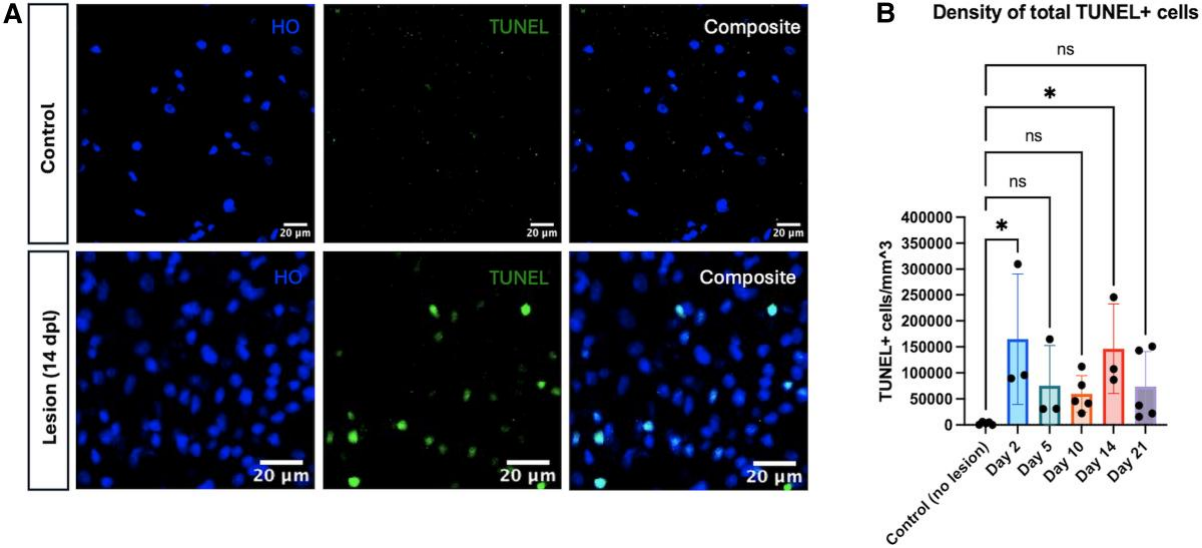
**Total TdT-mediated dUTP nick end labelling positive (TUNEL+) cell density remains elevated throughout the remyelination period**. (**A**) Representative immunofluorescence images of control and 14-day-old lesion tissue. Hoechst (HO, blue); TUNEL (green); composite (white). (**B**) The density of total TUNEL+ cells was quantified for each repair time point. Each individual data point represents the average across three regions of interest for a single animal. Bars represent mean ± SD. *P < 0.05, **P < 0.01, ***P < 0.001, and ****P < 0.0001 as determined by one-way ANOVA (control, *n* = 5; Day 2, *n* = 3; Day 5, *n* = 3; Day 10, *n* = 5; Day 14, *n* = 3; Day 21, *n* = 5), comparing each experimental group to the control group and applying Dunnett’s method for multiple comparisons. Abbreviation: dpl, days post-lesion.

TUNEL+ cells were observed at all post-lesion time points (Fig. 2A). The Day 2 time point featured an increased number of total TUNEL+ cells compared to baseline controls (*P* = 0.0172), as did the Day 14 time point (*P* = 0.0378) (Fig. 2B). This first peak of TUNEL+ cells corresponded to the period immediately following demyelinating injury (Day 2), while the second peak took place once remyelination was underway (Day 14).

### The densities of surviving and dying OLs remain stable throughout repair

**Figure 3:**
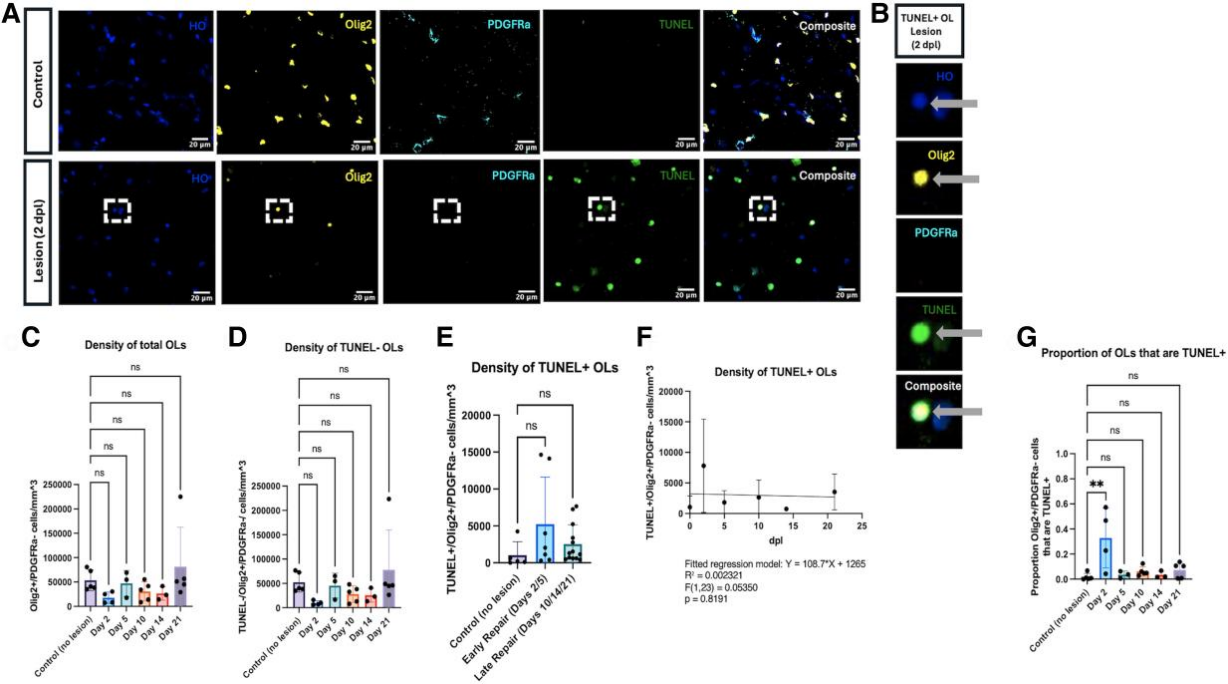
**The densities of surviving and dying OLs remain stable throughout repair.** (**A**) Representative immunofluorescence images of control and 2-day-old lesion tissue. Dashed box indicates cell shown in (**B**). Hoechst (HO, blue); Olig2 (yellow); PDGFRα (cyan); TUNEL (green); composite (white). (**B**) Representative immunofluorescence images of a single TUNEL+ OL. (**C**) The density of total OLs was quantified for each repair time point. (**D**) The density of TUNEL− OLs (‘surviving’ OLs) was quantified for each repair time point. (**E**) The density of TUNEL+ OLs was quantified for ‘early’ (Days 2/5) and ‘late’ (Days 10/14/21) repair. (**F**) Linear regression was used to model the density of TUNEL+ OLs over time. (**G**) The proportion of TUNEL+ OL was quantified for each repair time point. Each individual data point represents the average across three regions of interest for a single animal. Bars represent mean ± SD. *P < 0.05, **P < 0.01, ***P < 0.001, and ****P < 0.0001 as determined by one-way ANOVA (**C–D**, **G**; control, *n* = 5; Day 2, *n* = 4; Day 5, *n* = 3; Day 10, *n* = 5; Day 14, *n* = 3; Day 21, *n* = 5), comparing each experimental group to the control group and applying Dunnett’s method for multiple comparisons, or linear regression (**F**; control, *n* = 5; Day 2, *n* = 4; Day 5, *n* = 3; Day 10, *n* = 5; Day 14, *n* = 3; Day 21, *n* = 5). Abbreviation: dpl, days post-lesion.

Immunostaining for the oligodendroglial marker Olig2 and the OPC marker PDGFRα indicated the presence of both TUNEL+ oligodendrocytes (Olig2+/PDGFRα−) and OPCs (Olig2+/PDGFRα+) within the lesion boundary (Fig. 3A and B). There were no apparent differences across time points for the density of total OLs, TUNEL− OLs (‘surviving’ OLs), or TUNEL+ OLs (Fig. 3C–E). When linear regression was used to model the density of TUNEL+ OLs over time, no relationship was found between dpl and TUNEL+ OL density (Fig. 3F). The proportion of TUNEL+ OLs was increased at Day 2 compared to baseline (*P* = 0.0011) but was not increased at any other time points.

### Late remyelination time points are marked by an increased density of TUNEL+ OPC

**Figure 4:**
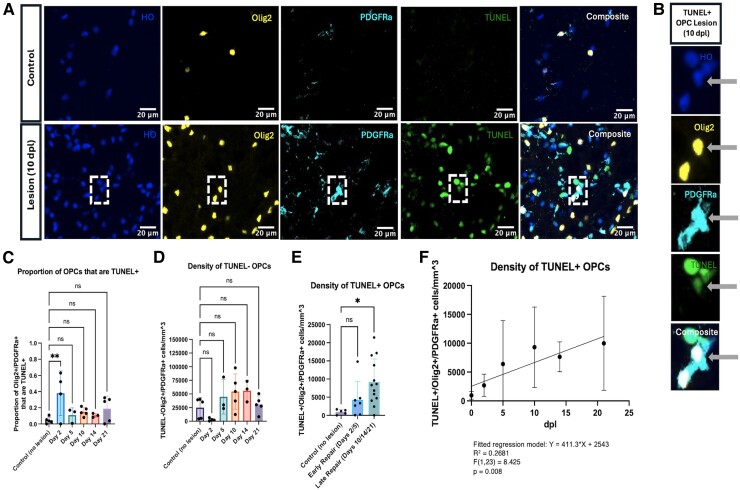
**Late remyelination time points are marked by an increased density of TUNEL+ OPC.** (**A**) Representative immunofluorescence images of control and 10-day-old lesion tissue. Dashed box indicates cell shown in (**B**). Hoechst (HO, blue); Olig2 (yellow); PDGFRα (cyan); TUNEL (green); composite (white). (**B**) Representative immunofluorescence images of a single TUNEL+ OPC. (**C**) The proportion of TUNEL+ OPCs was quantified for each repair time point. (**D**) The density of TUNEL− OPCs was quantified for each repair time point. (**E**) The density of TUNEL+ OPCs was quantified for ‘early’ (Days 2/5) and ‘late’ (Days 10/14/21) repair. (**F**) Linear regression was used to model the density of TUNEL+ OPCs over time. Each individual data point represents the average across three regions of interest for a single animal. Bars represent mean ± SD. *P < 0.05, **P < 0.01, ***P < 0.001, and ****P < 0.0001 as determined by one-way ANOVA (**C–E**; control, *n* = 5; Day 2, *n* = 4; Day 5, *n* = 3; Day 10, *n* = 5; Day 14, *n* = 3; and Day 21, *n* = 5), comparing each experimental group to the control group and applying Dunnett’s method for multiple comparisons, or linear regression (**F**; control, *n* = 5; Day 2, *n* = 4; Day 5, *n* = 3; Day 10, *n* = 5; Day 14, *n* = 3; and Day 21, *n* = 5). Abbreviation: dpl, days post-lesion.

Immunostaining for oligodendroglial markers Olig2 and PDGFRα indicated the presence of both TUNEL+ oligodendrocytes (Olig2+/PDGFRα−) and OPCs (Olig2+/PDGFRα+) within the lesion boundary (Fig. 4A and B). There was an increase in the proportion of OPCs that were TUNEL+ at Day 2 compared to baseline (*P* = 0.0077) (Fig. 4C). The density of TUNEL− OPCs (‘surviving’ OPCs) demonstrated no apparent differences across time points (Fig. 4D). The density of TUNEL+ OPCs, the remaining portion of OPCs, was increased compared to controls at late repair time points (Days 10, 14, and 21) (*P* = 0.0145) (Fig. 4E). When linear regression was used to model the density of TUNEL+ OPCs over time, the number of dpl predicted TUNEL+ OPC density (*P* = 0.008) (Fig. 4F).

### The majority of remyelination-associated TUNEL+ cells are Iba1+ microglia/macrophages

**Figure 5:**
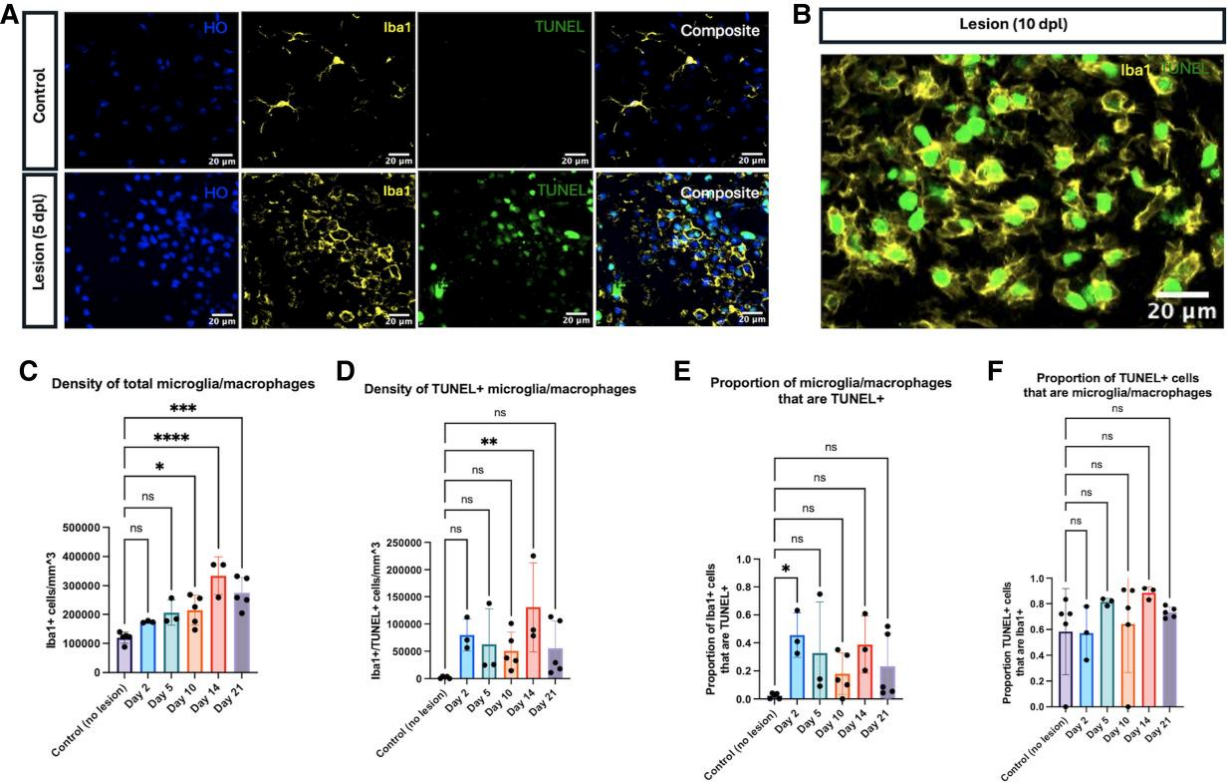
**The majority of TUNEL+ cells are Iba1+ microglia/macrophages.** (**A**) Representative immunofluorescence image of control and 5-day-old lesion tissue. Hoechst (HO, blue); Iba1 (yellow); TUNEL (green); composite (white). (**B**) Representative immunofluorescence image of TUNEL+ nuclei within Iba1+ microglia/macrophages from a 10-day-old lesion. (**C**) The density of total Iba1+ microglia/macrophages at each repair time point. (**D**) The density of total TUNEL+ microglia/macrophages at each repair time point. (**E**) The proportion of TUNEL+ microglia/macrophages (Iba1+ cells) at each repair time point. (**F**) The proportion of TUNEL+ cells that are Iba1+ microglia/macrophages at each repair time point. Each individual data point represents the average across three regions of interest for a single animal. Bars represent mean ± SD. *P < 0.05, **P < 0.01, ***P < 0.001, and ****P < 0.0001 as determined by one-way ANOVA (control, *n* = 5; Day 2, *n* = 3; Day 5, *n* = 3; Day 10, *n* = 5; Day 14, *n* = 3; Day 21, *n* = 5), comparing each experimental group to the control group and applying Dunnett’s method for multiple comparisons. Abbreviation: dpl, days post-lesion.

Immunostaining for Iba1+ cells demonstrated that most remyelination-associated TUNEL+ cells were Iba1+, indicating microglia and/or macrophages (Fig. 5A). High-resolution images of co-labelled TUNEL+/Iba1+ cells displayed TUNEL staining within Iba1+ microglia/macrophage nuclei, indicating DNA fragmentation within the nuclei of Iba1+ microglia/macrophages themselves, as opposed to cytoplasmic TUNEL staining more indicative of phagocytosed debris (Fig. 5B). The density of total microglia/macrophages was higher at late repair time points (Days 10, 14, and 21) compared to baseline control levels (control versus Day 10: *P* = 0.0165; control versus Day 14: *P* < 0.0001; control versus Day 21: *P* = 0.0002) (Fig. 5C). The density of TUNEL+ microglia/macrophages was elevated in lesioned animals compared to baseline controls; TUNEL+/Iba1+ cells were present at a significantly higher level compared to baseline at Day 14 (*P* = 0.0056) (Fig. 5D). The proportion of Iba1+ microglia/macrophages that were TUNEL+ was different than baseline at Day 2 (*P* = 0.0339) but demonstrated no apparent difference at later time points (Fig. 5E). The proportion of TUNEL+ cells that were Iba1+ microglia/macrophages did not differ from the baseline (mean = 0.5836) at any post-lesion time point (Fig. 5F).

## Discussion

According to the current model of successful CNS remyelination, following a demyelinating event, OPCs undergo recruitment to the lesion site, migration, proliferation, and differentiation into mature myelinating OLs.^4^ In this study, using a rodent model of toxin-induced demyelination, we found that lesion OPC counts at the late stages of remyelination did not differ from baseline levels, indicating that they were replenished following initial demyelinating injury. This finding aligns with the increased presence of proliferating OPCs observed during the early stages of remyelination (Days 2 and 5). Likewise, OL counts did not differ from baseline levels at late repair time points. Intriguingly, two peaks in overall cell death were observed in this study. The total number of TUNEL+ cells in the examined lesions was elevated at Day 2 following demyelinating injury—as expected due to the action of the toxin—and again at Day 14, once remyelination was underway. This late peak of TUNEL+ cells indicates that the repair period itself is characterized by a substantial level of cell death. Underlying this peak in total TUNEL+ cells, the density of TUNEL+ OPCs was increased in the late repair period (Days 10, 14, and 21) compared to control levels, indicating an increased presence of dying OPCs. However, TUNEL+ OPCs remained the minority of all OPCs, even at late stages of remyelination, and the minority of total TUNEL+ cells. The majority of TUNEL+ cells at all time points were Iba1+ microglia/macrophages.

While a formal mechanistic explanation is beyond the scope of this histology-based characterisation study, a working model of oligodendroglial and microglial cell death during successful remyelination can be formed based on these results. The glial cell dynamics captured in this study support the existence of a junction along an OPC’s progression to full differentiation at which the progenitor either proceeds to successfully differentiate into a myelinating OL or undergoes cell death. Based on the temporal patterns of glial cell density and turnover dynamics observed in this study, two different scenarios may be described. First, it may be the case that OPCs are migrating to the site of demyelination, proliferating to a point at which there are excess progenitors for the number of OLs required to repair the damage, and being pruned once differentiation has taken place. Second, OPCs may be responding to environmental cues and attempting to differentiate, but some portion is unable to successfully differentiate and undergoes cell death instead. This latter scenario of differentiation-associated progenitor cell death appears to be more likely, given that there was no decrease in the rate of OL differentiation observed during the late stages of repair.

Interestingly, the time point at which TUNEL+/Iba1+ microglia/macrophage density peaks (Day 14) overlaps with the peak TUNEL+ OPC density time point. These two events may be occurring independently or may be related. If they are related, it may be via interruption in the exchange of growth or survival factors between these glial cell populations occurring when one population undergoes extensive cell death, or via active release of apoptosis-promoting factors.^24^ Of course, it is also important to note that other CNS cell types with a role in remyelination, including astrocytes and vascular endothelial cells, were not characterized in this study.

Inflammation and repair often occur in close temporal proximity, likely due to the secretion of pro-remyelination factors by infiltrating immune cells, including microglia and macrophages.^25-28^ Indeed, previous work has described a critical window or ‘permissive period’ in which remyelination must occur in relation to inflammation in order to be successful, potentially due to the release of acute inflammatory stimuli promoting OPC maturation.^29-32^ Pro-remyelination properties of microglia have recently been identified beyond their roles in myelin debris clearance,^33,34^ including their role as drivers of OPC recruitment, proliferation, and OL differentiation.^35,36^ Rejuvenation of microglia/macrophages has also been shown to enhance remyelination in the aging CNS.^37^ Furthermore, necroptosis of pro-inflammatory microglia has been identified as a key driver of efficient CNS regeneration.^38^ Because the Iba1 antibody used in this study is not specific for microglia (it stains both microglia and macrophages), future work will be necessary to clarify the specific contributions of microglia and microglial subtypes, including pro-inflammatory and pro-regenerative microglia,^35^ to the overall levels of glial cell death associated with remyelination in this model. There remains uncertainty surrounding the factors contributing to the shift of microglia from a primarily pro-inflammatory phenotype to one promoting regeneration. It is possible that the death of OPCs during the late repair period could contribute to this transition, though this requires further mechanistic study, potentially through the use of apoptosis inhibitors or genetic manipulation. Additional studies focusing on later repair time points in this model would also help clarify the interesting finding that processes related to glial cell death and inflammation, including an increased density of total microglia/macrophages, persists beyond the final time point in our study.

While TUNEL staining is the most widely used assay for apoptotic cell death, it is not limited to detecting apoptosis-related DNA fragmentation. Other forms of cell death—including necrosis and necroptosis—may also appear TUNEL+.^39^ Further study is necessary to fully tease apart the individual contributions of different forms of cell death to glial cell turnover during remyelination in this model. Cells undergoing DNA repair may also appear TUNEL+. In this study, TUNEL+ OPCs and TUNEL+ microglia/macrophages peaked at the late stages of remyelination (Day 14), 2 weeks following induction with EB toxin. Due to this delay in becoming TUNEL+, and the fact that the vast majority of cells staining TUNEL+ must have migrated to the lesion site from elsewhere, it is unlikely that this TUNEL positivity is simply due to DNA repair. To this end, Ki67+/TUNEL+ OPCs were examined. While OPCs positive for both cell death and proliferation markers were increased at Day 2, they remained rare at all time points, with Ki67+/PDGFRα+ cells and TUNEL+/PDGFRα+ cells appearing as distinct populations (Supplementary Fig. S4). While a single marker (PDGFRα) was used to quantify OPCs in this staining panel, quantification of Olig2-/PDGFRα+ cells and total PDGFRα+ cells from another panel are provided for comparison in Supplementary Fig. S5.

**Figure 6:**
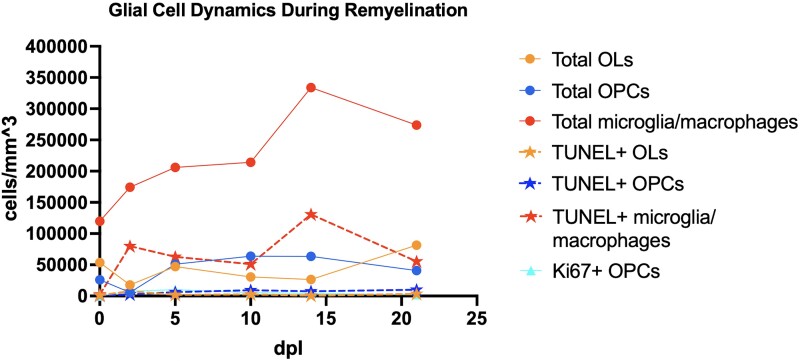
**Oligodendroglial and microglia/macrophage cell densities during remyelination in a toxin-induced model of demyelination.** TUNEL+ and Ki67+ subsets are shown. Datapoints represent averages across all animals at each time point (total OLs, *n* = 25; total OPCs, *n* = 25; total microglia/macrophages, *n* = 24; TUNEL+ OLs, *n* = 25; TUNEL+ OPCs, *n* = 25; TUNEL+ microglia/macrophages, *n* = 24; Ki67+ OPCs, *n* = 25). Standard errors are not shown to avoid figure cluttering but are available in Supplementary Table S2. Abbreviations: dpl, days post-lesion; OLs, oligodendrocytes; OPCs, oligodendrocyte progenitor cells.

This study increases our understanding of glial cell dynamics during the period surrounding remyelination (Fig. 6) and may provide the basis for future studies aimed at identifying molecular markers deriving from cells dying during the repair process. Proof-of-principle studies have previously show that OL-derived cfDNA can be detected in the serum of mice with cuprizone-induced demyelination as well as relapsing multiple sclerosis patients.^40,41^ As this study shows, elevated levels of glial cell death do indeed follow OPC proliferation, particularly in the later stages of remyelination. Therefore, it stands to reason that more OPC- or microglia-derived cfDNA, or other death-related by-products, would be released into the bloodstream in individuals undergoing increased levels of remyelination, such as those receiving remyelinating therapies in a clinical trial setting. Such a biomarker would have the advantage of arising from a cell-type not normally present in the blood (OPC/microglia), making it more specific to a neurological process. Candidate biomarkers could be identified and validated using recently published cell type-specific methylation atlases and ultrasensitive ddPCR-based approaches.^42,43^ Additional studies in the CCP-EB model using increased numbers of animals and focusing on later repair time points, combined with analysis of blood collected at those time points, may serve as the important link between the underlying CNS tissue repair processes and peripheral biomarkers necessary for investigating the translational potential of cell death-related by-products released during remyelination.

Regeneration in response to tissue injury is a vital homeostatic process. The apparent divide between globally destructive and reparative processes becomes less clear when we view each from the cellular level. In this study, we have shown that a generally reparative process, remyelination, intrinsically involves a high degree of glial cell death.

## Supplementary Material

fcae386_Supplementary_Data

## Data Availability

The data that support the findings of this study are available from the corresponding authors, upon reasonable request.
