# Supplementary material for: Significant oligodendrocyte progenitor and microglial cell death is a feature of remyelination following toxin-induced experimental demyelination
Source: Brain Commun. 2025 Jan 17;7(1):fcae386. doi: 10.1093/braincomms/fcae386 (PMC11739797; doi:10.1093/braincomms/fcae386)
Supplement: fcae386_Supplementary_Data [file fcae386_supplementary_data.zip › Supplementary_Figures.pdf]

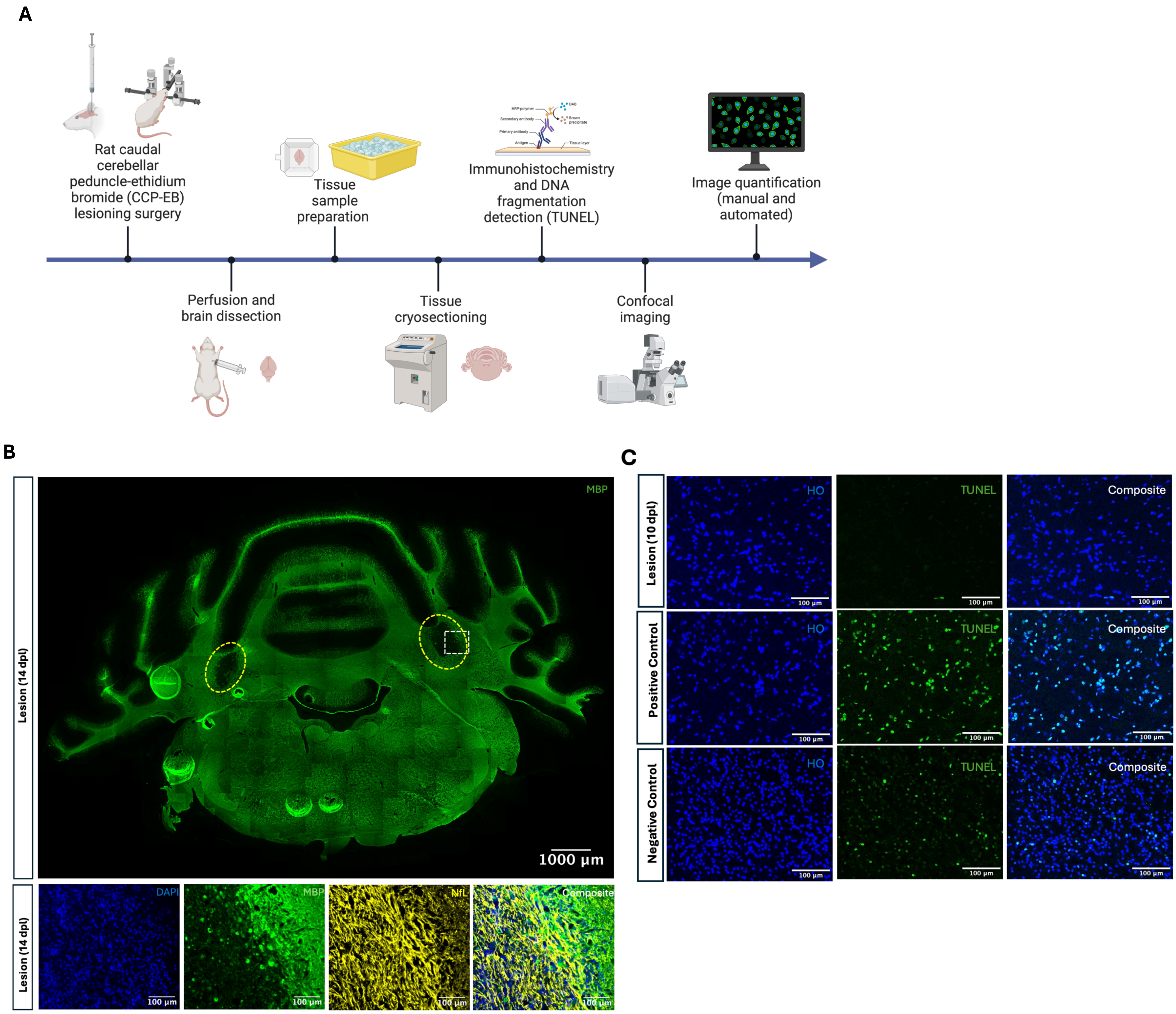

Supplementary Figure 1: Experimental outline, model characteristics, and TUNEL validation. (a) Experimental design for characterization of microglia/macrophage and oligodendroglial cell death and proliferation in a rodent model of toxin-induced demyelination. Perfusion and dissection were carried out at several post lesion timepoints (dpl; Control/Day 0, Day 2, Day 5, Day 10, Day 14, Day 21). (b) Toxin-induced demyelinated lesions were created in the caudal cerebellar peduncles (white matter tracts) using 4 µL 0.01% ethidium bromide. This toxin-induced rodent model of demyelination demonstrates near-complete myelin loss with preservation of axons. DAPI (blue); myelin basic protein (MBP, green); neurofilament light chain (NfL, yellow); composite (white). Yellow dashed ovals represent bilateral lesion locations. White dashed box represents zoomed-in region in bottom image panel (lesion edge). (c) Validation of the TUNEL assay using control and lesioned tissue. Caudal cerebellar peduncle from an unlesioned animal was used for a negative control. Positive controls were treated with DNAse I to induce DNA fragmentation. Images were taken at 20X magnification. Supplementary Figure 1A generated using BioRender.

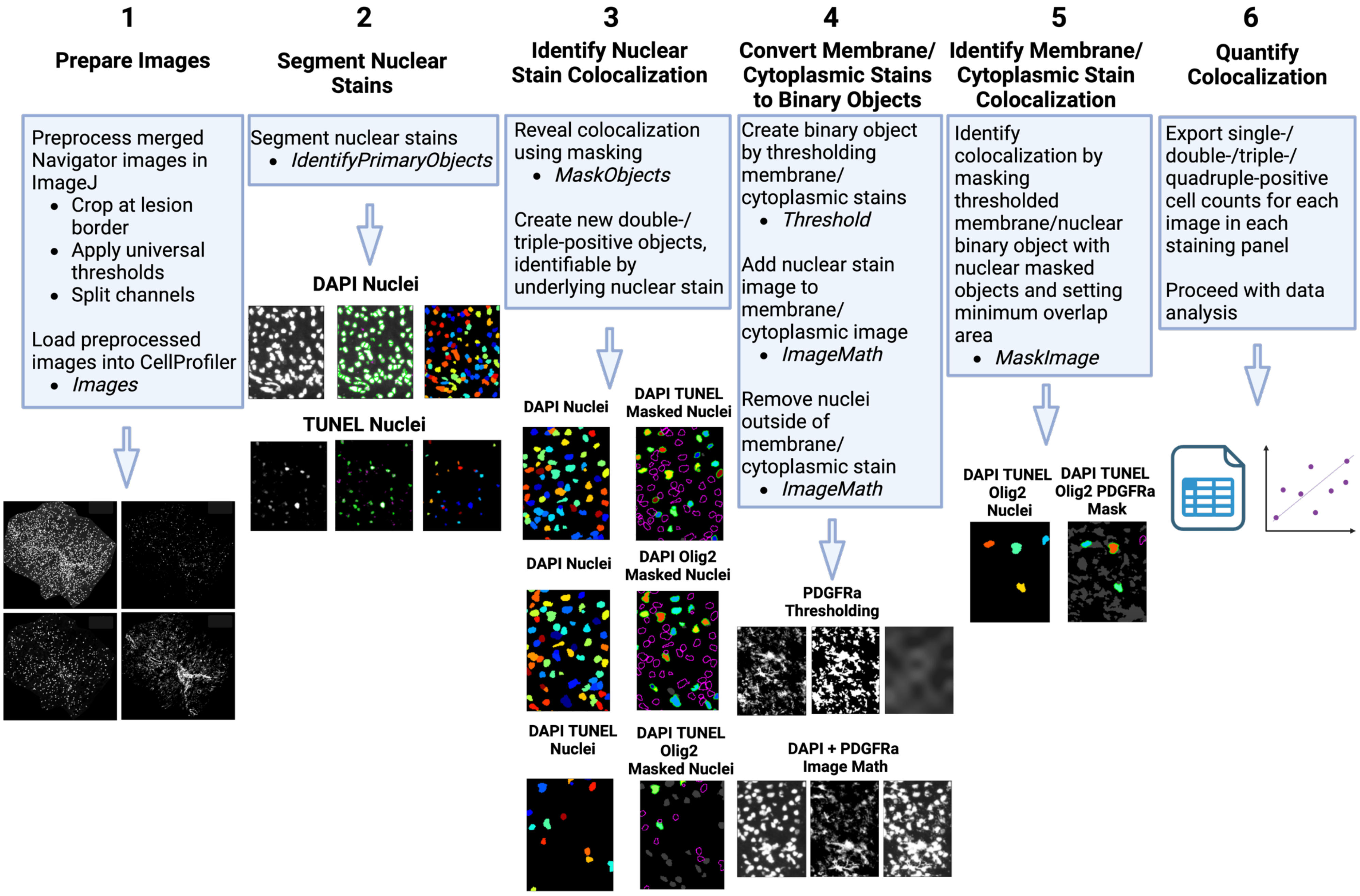

Supplementary Figure 2: CellProfiler pipeline general workflow. Custom CellProfiler pipelines were created to automatically quantify each staining panel. Merged confocal Navigator .lif files were preprocessed in Fiji/ImageJ to obtain separate channel images of the isolated lesion boundary with universal thresholds applied. Preprocessed images were loaded into CellProfiler and cells were segmented using nuclear stains (Hoechst/DAPI, TUNEL, Olig2, Ki67). Masks of each nuclear stain were overlaid to reveal colocalization, with each cell being traced back to its underlying DAPI stain boundary. Membrane and cytoplasmic stains (Iba1, PDGFRα) were binarized (foreground/background), then DAPI nuclei were added onto the image to fill in empty spaces and clarify cell borders. Nuclei outside of membrane/cytoplasmic stains were subtracted. Further colocalization identification was completed by overlaying thresholded binary images of membrane/cytoplasmic stains with nuclear object masks, with a universal minimum overlap area cutoff. Spreadsheets containing individual cell X/Y locations and staining characteristics were exported for further analysis. This figure was generated using BioRender.

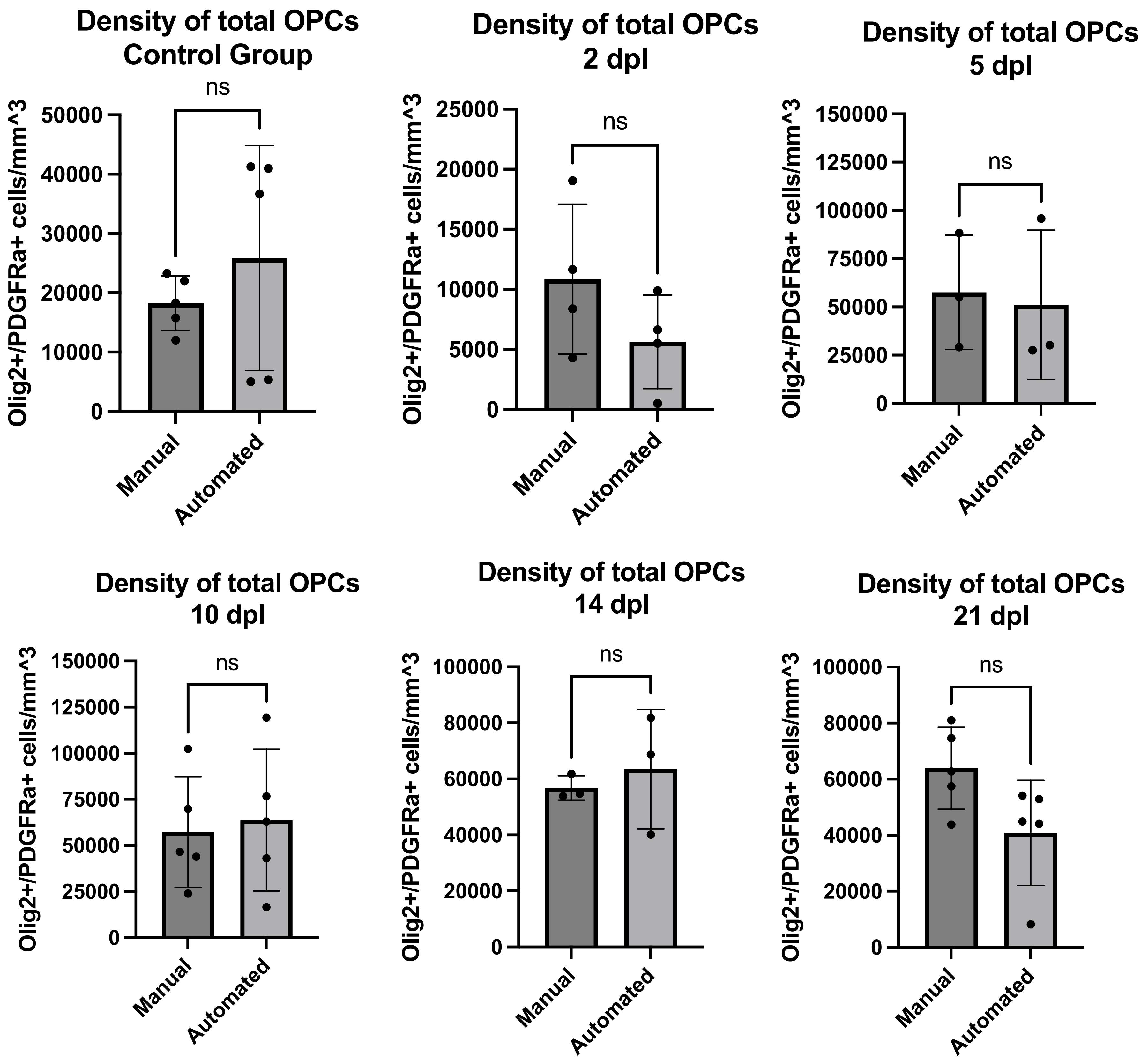

Supplementary Figure 3: Comparison of manual and automated co-localization quantification methods. Manual and automated cell counts for nuclear and membrane immunofluorescent oligodendroglial cell identity markers are comparable. Manual counts were completed using the Fiji/ImageJ Cell Counter plugin. Automated counts were completed using custom CellProfiler pipelines for each staining regimen. Each datapoint for manual counts represents one manual count of a single region of interest in a single animal, all counted by the same individual. Each datapoint for automated counts represents the average of automated counts for 3 regions of interest in a single animal. The same lesions from the same animals were counted for each quantification technique. A paired t-test was carried out (assuming consistent differences between paired values) for each timepoint (control, *n* = 5; Day 2, *n* = 4; Day 5, *n* = 3; Day 10, *n* = 5; Day 14, *n* = 3, Day 21, *n* = 5). The resulting two-tailed p-values for each timepoint were nonsignificant (>0.05). Bars represent means with standard deviations. Abbreviations: dpl = days post-lesion; OPC = oligodendrocyte progenitor cell.

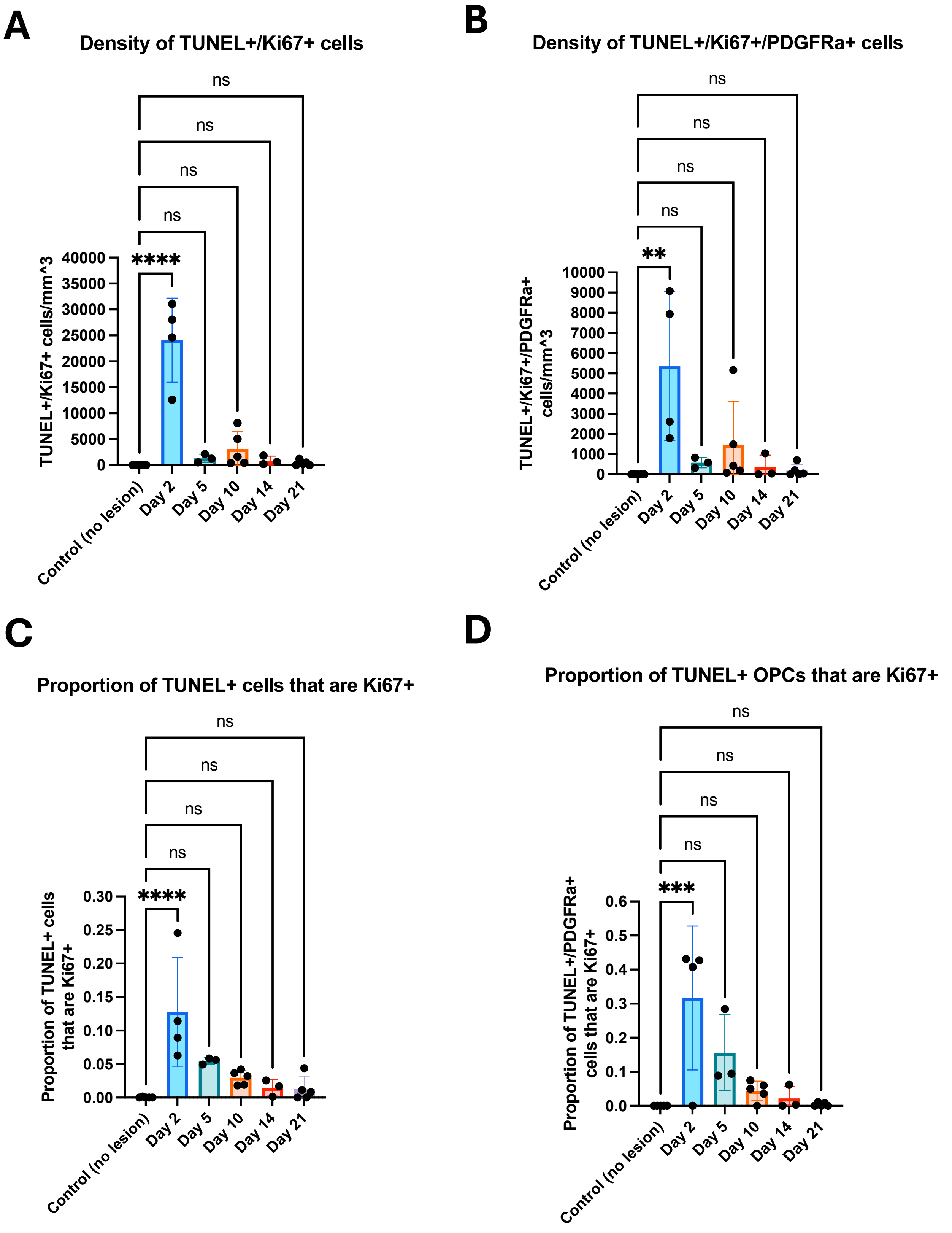

Supplementary Figure 4: Oligodendrocyte progenitor cells (OPCs) positive for cell death/proliferation markers are increased at Day 2 but remain rare. (a) The density of TUNEL+/Ki67+ cells was quantified for each repair timepoint. (b) The density of TUNEL+/Ki67+ OPCs was quantified for each repair timepoint. (c) The proportion of TUNEL+ cells that are Ki67+ was quantified for each repair timepoint. (d) The proportion of TUNEL+ OPCs that are Ki67+ was quantified at each repair timepoint. Each individual data point represents the average across three regions of interest for a single animal. Bars represent mean ± SD. *$p < 0.05$, **$p < 0.01$, ***$p < 0.001$, ****$p < 0.0001$ as determined by one-way ANOVA (c-e; control, n = 5; Day 2, n = 4; Day 5, n = 3; Day 10, n = 5; Day 14, n = 3, Day 21, n = 5), comparing each experimental group to the control group and applying Dunnett's method for multiple comparisons.

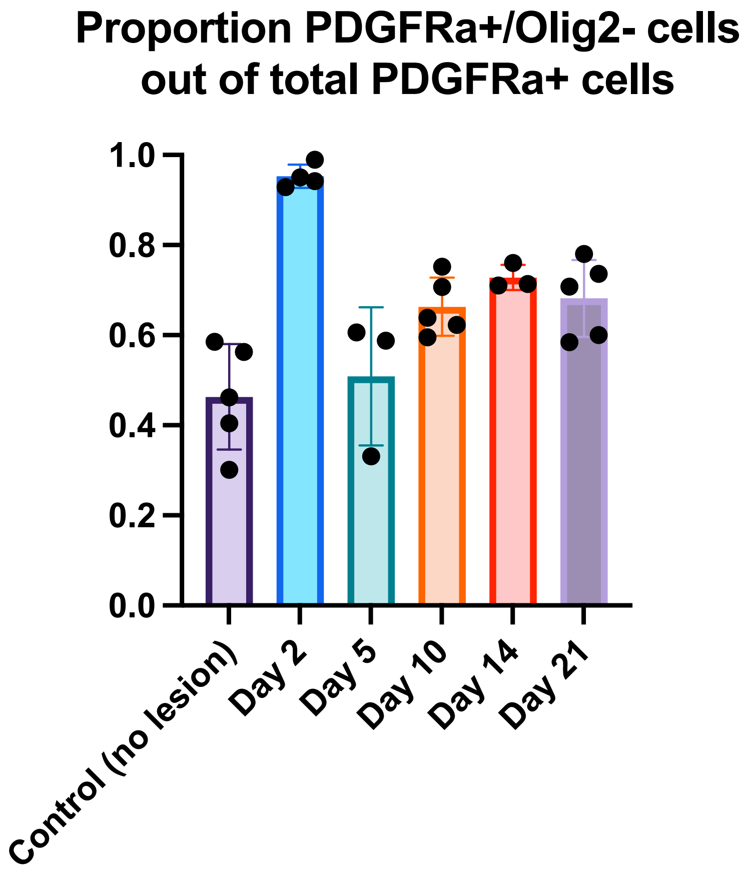

Supplementary Figure 5: A high proportion of the total PDGFRα+ cells segmented were not identified as colocalizing with Olig2. The proportion of PDGFRα+ cells that were PDGFRα+/Olig2- was quantified for each timepoint. Each individual data point represents the average across three regions of interest for a single animal (control, *n* = 5; Day 2, *n* = 4; Day 5, *n* = 3; Day 10, *n* = 5; Day 14, *n* = 3, Day 21, *n* = 5).
